# Ag Nanotwin-Assisted Grain Growth-Induced by Stress in SiO_2_/Ag/SiO_2_ Nanocap Arrays

**DOI:** 10.3390/nano8060436

**Published:** 2018-06-14

**Authors:** Fan Zhang, Yaxin Wang, Yongjun Zhang, Lei Chen, Yang Liu, Jinghai Yang

**Affiliations:** 1 Key Laboratory of Functional Materials Physics and Chemistry of the Ministry of Education, Jilin Normal University, Changchun 130103, China; zhangfan147258@126.com (F.Z.); liuyang@jlnu.edu.cn (Y.L.); jhyang1@jlnu.edu.cn (J.Y.); 2 College of Physics, Jilin Normal University, Siping 136000, China; 3 College of Chemistry, Jilin Normal University, Siping 136000, China; chenlei@jlnu.edu.cn

**Keywords:** SiO_2_/Ag/SiO_2_ nanocap arrays, grains growth, SERS

## Abstract

A trilayer SiO_2_/Ag/SiO_2_ nanocap array was prepared on a two-dimensional template. When annealed at different temperatures, the curvature of the SiO_2_/Ag/SiO_2_ nanocap arrays increased, which led to Ag nanocap shrinkage. The stress provided by the curved SiO_2_ layer induced the formation of Ag nanotwins. Ag nanotwins assisted the growth of nanoparticles when the neighboring nanotwins changed the local misorientations. Nanocap shrinkage reduced the surface plasmon resonance (SPR) coupling between neighboring nanocaps; concurrently, grain growth decreased the SPR coupling between the particles in each nanocap, which led to a red shift of the localized surface plasmon resonance (LSPR) bands and decreased the surface-enhanced Raman scattering (SERS) signals.

## 1. Introduction

Nanotwins, the plastic deformations of nanostructured materials, are widely reported among the face-centered cubic (fcc) metals [1,2,3]. Nanotwins have unique electrical conductivity [4,5], thermal stability [6,7], and sustained ductility [8,9,10]. The theory of thermodynamics and dynamics plays an important role in the formation of nanotwins [11]. Molecular dynamics (MD) simulation and experimental observations have revealed the relationship of nanotwins to the motion and dissociation of grain boundaries (GBs) [12]. GBs are the earliest known crystal defect, and the motion of GBs causes the grain growth. The driving forces for the migration of GBs include the grain boundary energy [13], chemical driving force [14], elastic energy [15], temperature gradient [16], et al. In comparison with traditional GBs, the twin boundaries (TBs) have higher symmetry and lower energy. The Σ3{111} coherent twin boundaries in the nanotwins have very low energy; thus, the nanotwins are stable at high temperature [17]. GBs are usually unstable when samples are induced mechanically, which leads to GB migration [18]. In addition to mechanically induced stress, stress in the curved nanostructures was also observed, which led to the typical evolution of the physical properties [19]. This type of stress can be adjusted by the curvature and the film thickness.

Surface plasma is the collective movement of electrons in metals, which occurs when the electrons are driven by an electromagnetic wave [20]. When the fluctuation of the surface plasma-formed field is no longer continuous in space, for example, in isolated metal nanostructures, the surface plasmon becomes a localized surface plasmon (LSP). Surface plasmon excited by light requires a degree of surface roughness or curvature [21,22]. If the morphology of the samples or the nanoparticles in the nanostructures is changed by the stress, the localized surface plasmon resonance (LSPR) performance will be tuned [23,24,25].

Herein, we prepared PS 500 nm/SiO_2_ 12 nm/Ag 60 nm/SiO_2_ 12 nm nanocap arrays, in which the curved SiO_2_ layer supplied consistent stress to the Ag layer. By controlling the annealing temperature, the curvature of the SiO_2_/Ag/SiO_2_ nanocap arrays as well as the stress increased. Stress induced the grain rotation and formation of Ag nanotwins in SiO_2_/Ag/SiO_2_ nanocap arrays when annealed. The GB dissociation and grain growth were also observed at different temperatures. The morphology and microstructure changes led to changes in the surface plasmon.

## 2. Experimental Section

### 2.1. Materials

Monodisperse polystyrene (PS) colloid particles have a density of 1.05 g/cm^3^, with an average diameter of 500 nm; they were purchased from the Duke Scientific Corporation. 4-Mercaptobenzoic acid (MBA, 99%) and sodium dodecyl sulfate (analytic reagent, AR), NH_4_OH (25%), and H_2_O_2_ (30%) were purchased from the Sigma-Aldrich Co., Ltd. (St. Louis, MO, USA) and the Sinopharm Chemical Reagent Co., Ltd. (Shenyang, China).

Ag and SiO_2_ targets purity were 99.99%, and the silicon wafer with (100) crystal orientation, which were purchased from the Beijing Jing Mai Mstar Technology Co., Ltd. (Beijing, China) and the Hefei Kejing Materials Technology Co., Ltd. (Hefei, China), respectively. The resistivity of de-ionized (DI) water was 18.0 Mcm^−1^ and ethanol (AR) was used in the whole process of preparation.

### 2.2. Preparation of PS Colloidal Sphere Arrays

We prepared the PS colloidal sphere arrays with size of 500 nm by the self-assembly technique. First, the silicon wafer was cut into desired sizes and placed in a H_2_O, NH_4_OH, and H_2_O_2_ (volume ratio 6:1:2) solution, followed by boiling at 300 °C for approximately 5–10 min. The solution was then poured out and sonicated in DI water and alcohol three times. Subsequently, the Si wafer was stored in DI water until use. Second, 10% PS colloidal spheres were mixed with alcohol and DI water. The mixture was introduced into a Petri dish with DI water through a tube. Small spheres were seen floating on the water surface. When the spheres were covered with water, the water was replaced several times to ensure the formation of monolayer spheres. After draining, the monolayer spheres were formed on the Si wafer.

### 2.3. Preparation and Annealing of PS 500 nm/SiO_2_ 12 nm/Ag 60 nm/SiO_2_ 12 nm Nanocap Arrays

The magnetron sputtering system was used to prepare SiO_2_ and Ag films. Ar was used as the sputter gas, and the sputter chamber pressure was 0.6 Pa. The SiO_2_ film thickness was 12 nm, while the sputter power was 100 W. The Ag film sputter power was 21.6 W, and the Ag film thickness was 60 nm. The SiO_2_/Ag/SiO_2_ nanocap arrays were dipped into tetrahydrofuran to remove the colloidal sphere arrays. The samples were then cleaned with alcohol. The samples without the colloidal spheres were annealed by Ar.

### 2.4. Characterization of Substrates

The performance characterization methods used in the experiment predominantly included the scanning electron microscope (SEM), transmission electron microscope (TEM), UV-Vis spectra with a Shimadzu UV-3600 spectrophotometer (Shimadzu, Kyoto, Japan), and X-ray powder diffraction (XRD). Analyses were conducted using a Rigaku D/MAX 3C X-ray diffractometer with Cu Kα radiation (λ = 1.54060 Å), and Raman spectra were measured on a Renishaw Raman confocal microscopy spectrometer (model 2000, Renishaw, London, UK). The laser power was 40 mW, and the excitation wavelength was 514.5 nm. A tube furnace (OTF-1200X, Hefeikejing, Hefei, China) was used for the annealing. The heating rate was 5 °C/min.

## 3. Results and Discussion

**Figure 1:**
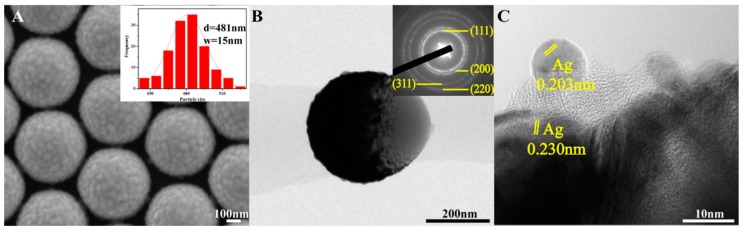
(**A**) SEM, (**B**) TEM, and (**C**) HRTEM images of a SiO_2_/Ag/SiO_2_ nanocap arrays. The insets are the size distribution and the Ag SAED pattern.

The SiO_2_/Ag/SiO_2_ nanocap arrays were in a compact periodic arrangement, and the average distance between the neighboring nanocaps was approximately 20 nm. The average lateral size of the nanocap was approximately 480 nm, and the standard deviation was approximately 15 nm. When Ag was deposited onto the SiO_2_ layer, Ag tended to form round particles because the SiO_2_ layer does not have good compatibility with Ag. Therefore, the nanocap surface was rough, and many small protruding parts were observed, as shown in Figure 1A. Figure 1B shows the SiO_2_/Ag/SiO_2_ nanostructure TEM images, and the SiO_2_/Ag/SiO_2_ trilayer covered the PS sphere completely, confirming the formation of the nanocap. The selected area electron diffraction (SAED) showed Ag with a polycrystalline structure, and an amorphous material was observed over Ag, which is believed to be SiO_2_. The high-resolution transmission electron microscopy (HRTEM) image in Figure 1C shows that the distances of the lattice fringes are approximately 0.230 and 0.204 nm, which agrees with the (200) and (111) planes of the Ag well [26].

**Figure 2:**
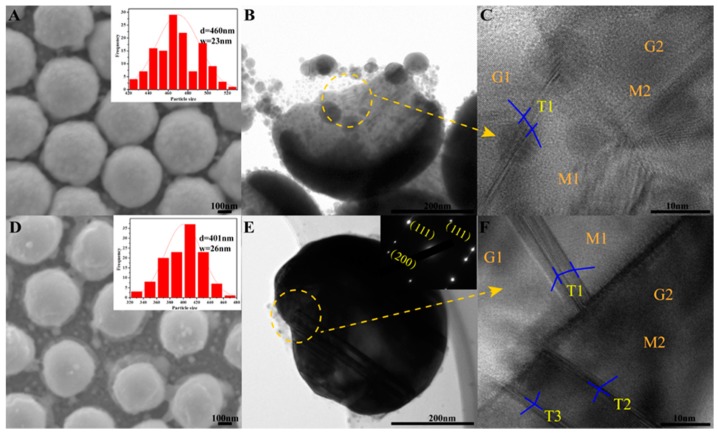
(**A**) SEM, (**B**) TEM and (**C**) HRTEM images of SiO_2_/Ag/SiO_2_ nanocap arrays annealed at 400 °C and (**D**–**F**) 800 °C. The inserts are the fast Fourier transform (FFT) of the Ag nanotwins; T, G, and M are the abbreviations for twin, grain, and matrix.

When the trilayer film was annealed at 400 °C, the nanocaps maintained their complete morphology, indicating that the SiO_2_/Ag/SiO_2_ nanocap arrays have good thermal stability. The nanocaps showed a little shrinkage; the average size of the nanocaps was 460 nm, with a standard deviation of 23 nm, which indicates that the SiO_2_ layer has good mechanical properties for maintaining the shape of the nanocaps in Figure 2A. Each nanocap was completely isolated, and the surface of the nanocap became smooth. The amount of the protruding parts decreased in each nanocap during the annealing process, indicating that the melting process occurred in each nanocap. The TEM image shows that the range of the grain sizes in the nanocaps was 5~30 nm after annealing at 400 °C, which confirms that the grain growth happened in the nanocaps when annealed. The HRTEM in Figure 2C shows the nanotwins in the annealed nanocaps. In grain 1 (G1) and grain 2 (G2), the misorientation of matrix 1 (M1) and matrix 2 (M2) is 73.5°. After twin 1 (T1) formed in G1, the angle between M1 and M2 was 90.2°. The nanotwins impinged on the GBs and then changed the local misorientation of adjacent grains. When annealed at 800 °C, the nanocaps showed obvious shrinkage, and the lateral size observed was 400 nm. The shrinkage of the SiO_2_ layer indicated the larger stress in the nanocaps when annealed at 800 °C. The surface of the nanocaps was very smooth and no additional or obvious nanoparticles or protruding parts were observed in Figure 2D. Figure 2E shows that the amorphous brim part was transparent on each nanocap, which is believed to be the supporting SiO_2_ frame. The grain size range in the nanocaps was 10~60 nm. The fast Fourier transform (FFT) of the Ag nanotwins proved the existence of Σ3{111} coherent twin boundaries. At 800 °C, T1, T2, and T3 were observed in grains G1 and G2. T1 changed the local misorientation of G1 to the same orientation as G2, which helped G1 and G2 grow together.

The grain size is an important factor affecting the deformation twins of metals and alloys, which creates the local stress that contributes to the twin nucleation and grain growth [27,28]. According to Hall–Petch type (H–P) behavior [29], the critical stresses for dislocation slip and twinning is represented by δ, $\delta =\delta_{0}+kd^{-1/2}$, where *d* is the grain size and *k* is the constant. For fcc metals, when the grain sizes increases, the critical stress decreases rapidly, leading to dislocation slip. Thus, the larger grain sizes are more favorable for twin nucleation. Experimental observations have revealed that nanotwins promote GB dissociation and grain growth. The local dissociation of the GB of two adjacent grains results in a high-angle GB turning into a low-angle GB. Subsequently, the low-angle GB disappears; as a result, the lattices turn to the same angle and two adjacent grains form a larger grain [12].

For the as-deposited samples, the local stress was small and was not enough for nanotwin formation. During the annealing process, the curvature of the SiO_2_/Ag/SiO_2_ nanocap arrays continually increased, which resulted in the larger local stress. The Stoney formula [30,31] for calculating the film stress is as follows:

$$\delta =(\frac{E}{1-\gamma })\frac{t_{s}{}^{2}}{6t_{f}}\cdot \frac{1}{R},$$

where δ is the SiO_2_ film stress, *E* is Young’s modulus, *γ* is the Poisson ratio of substrate, *t_s_* and *t_f_* are thicknesses of the substrate and film, respectively, and *R* is curved radius of the substrate curvature. When annealed at temperatures of 400 °C and 800 °C, both the film curvature and the stress increased.

According to the Coble creep equation [32], the creep rate ε, can be calculated in the presence of both stress and temperature as follows:

$$\varepsilon =\frac{A}{d^{3}}\cdot \frac{A}{T}e^{-H_{b}/RT},$$

where *A* is a constant, δ is the critical stress to forming nanotwins, *d* is the average grain size, *H_b_* is the activation energy, *R* is the gas constant, and *T* is the temperature. This shows that stress and temperature promote grain growth and are the joint reason behind the larger grains at 800 °C compared to those at 400 °C.

**Figure 3:**
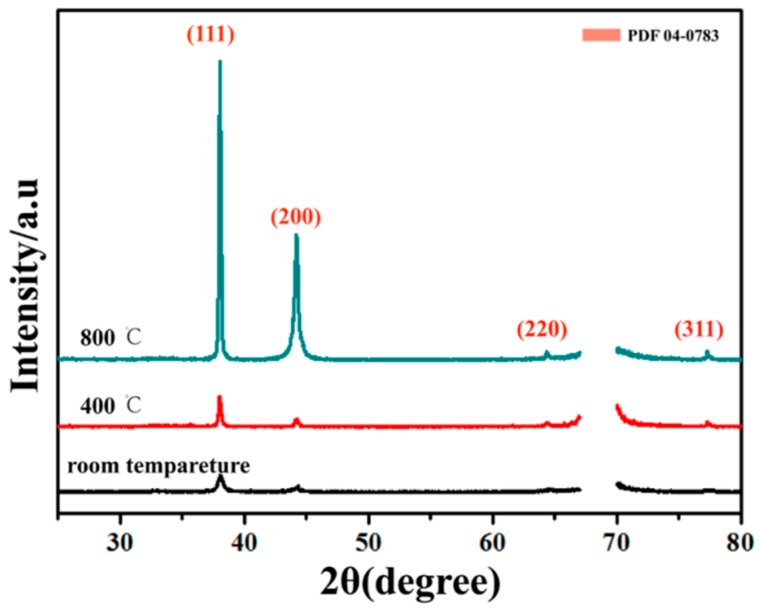
XRD patterns of the SiO_2_/Ag/SiO_2_ nanocap arrays at different temperatures.

XRD was also used to observe the formation of Ag nanotwins in the SiO_2_/Ag/SiO_2_ nanocap arrays. In Figure 3, the SiO_2_/Ag/SiO_2_ nanocap arrays retain a distinct diffraction peak at all temperatures, indicating that the SiO_2_/Ag/SiO_2_ nanocap arrays were also very stable at high temperatures. No new phases were found in the XRD, which indicated that high temperatures do not destroy the Ag crystal structure. No SiO_2_ peak was observed, illustrating SiO_2_ to be amorphous. The data displayed diffraction peaks at 2θ = 77.5°, 64.4°, 44.3°, and 38.1°, which could be indexed to the (311), (220), (200), and (111) planes of the fcc Ag, respectively [33]. When annealed, all the full width at half-maximum (FWHM) values of the (111) and (200) diffraction peaks of Ag narrowed, illustrating that the grain size had increased. With the increase of the annealing temperature, the diffraction peaks of the (111) and (200) planes of Ag grew stronger, showing that the SiO_2_/Ag/SiO_2_ nanocaps have better crystallinity [34].

**Figure 4:**
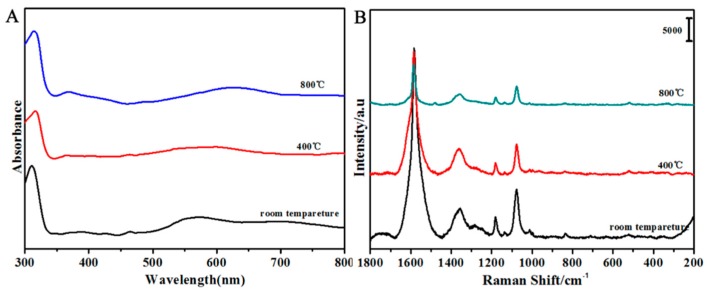
UV-Vis absorbed spectra (**A**) and SERS spectra (**B**) of the SiO_2_/Ag/SiO_2_ nanocap arrays at different temperatures.

UV-Vis spectroscopy was used to monitor the evolution of the SiO_2_/Ag/SiO_2_ nanocap arrays during the annealing process. As shown in Figure 4A, the absorption peak at approximately 320 nm did not change with temperature variation, which originated from Ag band transitions [35]. At room temperature, the SiO_2_/Ag/SiO_2_ nanocap arrays possessed two transverse broad peaks at approximately 500–800 nm, which were assigned to Ag local surface plasmon resonance (LSPR) bands. As the temperature increased, Ag LSPR bands gradually red shifted, which originated from the Ag grain size increase because LSPR bands are very sensitive to the size and the aspect ratio [36]. When annealed, LSPR bands broadened because Ag nanotwins made the adjacent grains combine into a larger one.

In Figure 4B, the 4-MBA probe molecules were absorbed on SiO_2_/Ag/SiO_2_ nanocap arrays. The spectrum shows the intense SERS obvious characteristic peaks of 4-MBA, which was dominated by the bands at 1077 and 1584 cm^−1^ assigned to υ(CC) ring-breathing modes and the intense mode, including δ(CH)(1182 cm^−1^ and 1133 cm^−1^), υ(CC)(COO-)(1362 cm^−1^), and υ(CC) + α(CH) combination modes (1481 cm^−1^) of Raman spectra [37,38]. When the temperature increased, the SERS intensity decreased. Assignments of the observed Raman bands are summarized in Table 1. The intensity of the characteristic peak at 1584 cm^−1^ decreased with the increase of the annealing temperature. There are two reasons for the SERS signal decrease. As the temperature increased, the gap between the nanocaps gradually became larger, which led to weakened couplings between the neighboring nanocaps. When the samples were annealed at 400 °C, the surface of the nanocap became smooth; the Ag grain growth reduced the coupling between the particles in the nanocaps, which led to the decreased “hot spot” in each nanocap [39,40]. At 800 °C, the SERS intensity was the lowest, but the enhancement factor (EF) was about 10^7^. Even at high temperatures, the SiO_2_/Ag/SiO_2_ nanocap arrays had the SERS enhancement.

## 4. Conclusions

The SiO_2_/Ag/SiO_2_ nanocap arrays were prepared by magnetron sputtering on two-dimensional PS arrays. After annealing at 400 and 800 °C, the SiO_2_/Ag/SiO_2_ nanocaps shrank, which resulted in the growth of the nanogap size between the neighboring nanocaps. Simultaneously, after the annealing, the nanoparticles in the nanocaps grew into large grains due to the formation of nanotwins, which was caused by the stress. Grain boundary (GB) dissociation and grain rotation also promoted grain growth. Additionally, the unique SiO_2_/Ag/SiO_2_ nanotwin structures exhibited good thermal stability and excellent surface-enhanced Raman scattering (SERS) properties.

## Figures and Tables

**Table 1:** Raman band frequencies of 4-MBA.

Wavenumber (cm^−1^)	Band Assignment
1584	υ(CC) ring
1481	υ(CC) + δ(CH)
1360	νs(COO-)
1182	δ(CH)
1133	δ(CH)
1077	υ(CC) ring

(δ) bend or deformation; (υ) stretch; (ring) ring breathing mode; (α) antisymmetric.
